# Supplementary material for: Cement augmentation for trochanteric femur fractures: A meta-analysis of randomized clinical trials and observational studies
Source: PLoS One. 2021 Jun 15;16(6):e0251894. doi: 10.1371/journal.pone.0251894 (PMC8205169; doi:10.1371/journal.pone.0251894)

# S1 File

**Cement augmentation for trochanteric femur fractures: a meta-analysis and systematic review of randomized clinical trials and observational studies.**

##

## S1 Table: Search syntax

| Database | Syntax |
| --- | --- |
| **Pubmed/MEDLINE (n=997)** | (((cement*[Title/Abstract]) OR (augment*[Title/Abstract])) OR (reinforcemen*[Title/Abstract])) AND ((((((intertrochanteric[Title/Abstract]) OR (pertrochanteric[Title/Abstract])) OR (trochanteric[Title/Abstract])) OR (proximal femur frac*[Title/Abstract])) OR (femoral neck frac*[Title/Abstract])) OR (collum frac*[Title/Abstract])) |
| **Embase (n=544)** | ('cement'/exp OR cement OR augment:jt OR reinforcement:au) AND (trochanteric:ti,ab,kw OR subtrochanteric:ti,ab,kw OR intertrochanteric:ti,ab,kw OR pertrochanteric:ti,ab,kw OR 'proximal femure fracture':ti,ab,kw OR 'femoral neck fracture':ti,ab,kw OR 'collum fracture':ti,ab,kw) |
| **CENTRAL (n=50)**  **CINAHL (n=247)** | ('cement'/exp OR cement OR augment:jt OR reinforcement:au) AND (trochanteric:ti,ab,kw OR subtrochanteric:ti,ab,kw OR intertrochanteric:ti,ab,kw OR pertrochanteric:ti,ab,kw OR 'proximal femure fracture':ti,ab,kw OR 'femoral neck fracture':ti,ab,kw OR 'collum fracture':ti,ab,kw) |
|  |  |

S2 Table: Quality assessment criteria

| **Criteria** | **Reported and adequate (2)** | **Reported but inadequate (1)** | **Not reported (0)** |
| --- | --- | --- | --- |
|  |  |  |  |
| Clearly stated aim | Aim including outcomes reported | Aim reported without outcomes | Not reported |
| Inclusion consecutive patients | Inclusion/exclusion criteria reported | Unclear description inclusion/exclusion criteria | Not reported |
| Prospective collection data | Prospective | retrospective | Not applicable |
| Appropriate endpoints | Appropriate endpoints to aim study | Endpoints not appropriate to aim study | Not reported |
| Unbiased assessment | Blinded evaluation of outcomes | Reason not blinding stated | Not reported |
| Appropriate follow-up | ≥ 1 year | < 1 year | Not reported |
| Loss to follow-up < 5% | ≤ 5% | > 5% | Not applicable |
| Prospective calculation study size | Prospective power-analysis performed | Prospective calculation without power-analysis | Not applicable |
| Adequate control group | Operative versus nonoperative treatment | Not applicable | Not applicable |
| Contemporary groups | Study/control group managed during same period | Study/control not managed during same period | Not reported |
| Baseline equivalence groups | Baseline characteristics described and comparable | Baseline characteristics not comparable | Not reported |
| Adequate statistical analyses | Statistical analysis described including type of analyses | Inadequate description statistical analysis | Not reported |

## S3 Table: Quality assessment

|  | **RCT** | Kammerlander 2018 | Dall oca 2010 | Mattson 2004 | Lee 2009 | **Observational Studies** | Yee 2020 | Kim 2018 | Kulachote 2019 |
| --- | --- | --- | --- | --- | --- | --- | --- | --- | --- |
|  |  |  |  |  |  |  |  |  |  |
| Clearly stated aim |  | 2 | 2 | 2 | 2 |  | 2 | 2 | 2 |
| Inclusion of consequetive patients |  | 1 | 2 | 2 | 2 |  | 2 | 2 | 2 |
| Prospective data collection |  | 2 | 2 | 2 | 2 |  | 1 | 1 | 1 |
| Appropriate endpoints |  | 2 | 1 | 1 | 2 |  | 2 | 2 | 2 |
| Unbiased assessment endpoints |  | 1 | 0 | 0 | 0 |  | 0 | 0 | 0 |
| Appropriate follow-up (>1year) |  | 2 | 2 | 1 | 2 |  | 1 | 1 | 2 |
| Loss-to-follow-up <5% |  | 1 | 1 | 1 | 1 |  | 1 | 1 | 1 |
| Prospective calculation study size |  | 2 | 1 | 2 | 2 |  | 2 | 1 | 2 |
| Adequate control group |  | 2 | 2 | 2 | 2 |  | 2 | 2 | 2 |
| Contemporary groups |  | 2 | 2 | 0 | 2 |  | 2 | 2 | 1 |
| Baseline quivalence of groups |  | 2 | 2 | 2 | 2 |  | 2 | 2 | 1 |
| Adequate statistical analysis |  | 2 | 2 | 2 | 2 |  | 2 | 2 | 2 |
| **Total:** |  | **21** | **19** | **17** | **21** |  | **19** | **18** | **18** |

## S4 Table: Other baseline characteristics

| **Author** | **Reduction quality augmentation** | **Reduction quality no augmentation** | **Blade position intraop augmentation** | **Blade position intraop no augmentation** | **smoker (%)** | **diabetes (%)** |
| --- | --- | --- | --- | --- | --- | --- |
| **RCT** |  |  |  |  | augmented/control | augmented/control |
| Kammerlander | poor intraoperative fracture reduction n=0 | poor intraoperative fracture reduction n=1 | TAD 26.9 (SD1.5) | TAD 24.2 (SD1.2) | 12. Jul | nr |
| Dall oca | nr | nr | nr | nr | nr | nr |
| Lee | all acceptable* | all acceptable | Middle 1/3 (n=22), Lower (n=33) | middle 1/3 (n=19), Lower (n=34) | nr | nr |
| Mattson | nr | nr | nr | nr | nr | nr |
| **Observational studies** |  |  |  |  |  |  |
| Yee | Good (80.9%, n=38), Acceptable (19.1%, n=9), Poor 0 | Good (79.3%, n= 23), Acceptable (20.7%, n= 6), Poor (0) | TAD 18.4 (SD 4.9) | TAD 20.3 (5.6) | nr | nr |
| Kulachote | nr | nr | center position 65% (n=44) | center position 70% (47n) | nr | 35/37.3 |
| Kim | nr | nr | adequate (93%), TAD 19mm (SD2) | adequate (90%), TAD 17mm (SD2) | nr | nr |

*(anatomic or slight valgus alignment on the anteroposterior (AP) view, alignment with parallel or slight cervical anteversion on the lateral view, and no more than 1 cm of displacement between two major fracture fragments). nr=not reported

## S5 Table: Inclusion and exclusion criteria

| **Author** | **Study group** | **Exclusion Criteria** |
| --- | --- | --- |
| **RCT** |  |  |
| Kammerlander | >75y, AO Type 31 A2-A3, low energy trauma, OP within 72h after admission, walking ability | pathologic fx, open fx., polytrauma, hemiplegia, implant in same hip, wire penetration into hip joint, hypersensitivity/allergy to PMMA |
| Dall oca | A2.1, 2.2, 2.3, & A3, >80, Sigh scores 1 or 2 | dementia, neuromusc deficiency, malignancy, polytrauma, corticosteroid therapy |
| Lee | >75y, AO Type 31 A2-A3, low energy trauma | stable fractures, open fractures, pathologic fractures, previous ipsilateral hip surgery, developmental abnormaility |
| Mattson | AO-A2, low energy trauma (same height fall), walking ability, normal contralateral hip | pathologic fx, polytrauma |
| **Observational studies** |  |  |
| Yee | >65y, AO 31A, low energy trauma (ground level fall) | pathologic fracture, delayed fixation, hip disaeses other than mild osteoarthritis, preexisting other implants |
| Kulachote | >70, AO 31 A1,2 & 3, low energy trauma, ASA 3 & 4, walking ability | pathologic fx, polytrauma,, open fracture, active cancer, allergy to bone cement |
| Kim | AO A2-3, osteoporosis <2.5 (t-Score) | polytrauma, stable fractures, DHS |

## S6 Table: List of complications

| **Complications** | **Augment-tation** | **(%)** | **No augmentation** | **(%)** |
| --- | --- | --- | --- | --- |
| **Systemic** | **73** | **47% (n=155)** | **105** | **52% (n=202)** |
| Thromboembolic | 6 | 3.9 | 1 | 0.5 |
| Delirium | 5 | 3.2 | 18 | 8.9 |
| Pneumonia | 6 | 3.9 | 11 | 5.4 |
| Stroke | 3 | 1.9 | 10 | 5.0 |
| BCIS | 1 | 0.6 | 0 | 0.0 |
| Other systemic | 52 | 33.5 | 65 | 32.2 |
|  |  |  |  |  |
| **Device related/local** | **18** | **6% (n=297)** | **65** | **20% (n=326)** |
| Superficial wound infection | 5 | 1.7 | 8 | 2.5 |
| Deep wound infection | 1 | 0.3 | 1 | 0.3 |
| Cement leakage | 2 | 0.7 | 0 | 0.0 |
| Fixation failure | 1 | 0.3 | 4 | 1.2 |
| Loosening/excessive screw/blade sliding | 0 | 0.0 | 18 | 5.5 |
| Bending/breaking | 2 | 0.7 | 1 | 0.3 |
| Cut out or cut through | 0 | 0.0 | 10 | 3.1 |
| Delayed union | 0 | 0.0 | 0 | 0.0 |
| Nonunion | 0 | 0.0 | 0 | 0.0 |
| Malunion | 2 | 0.7 | 23 | 7.1 |
| Refracture | 0 | 0.0 | 1 | 0.3 |
| Periimplant fracture | 1 | 0.3 | 3 | 0.9 |
| Irritation of tractus iliotibialis | 0 | 0.0 | 3 | 0.9 |
| Hematoma requiring re-intervention | 1 | 0.3 | 3 | 0.9 |
| Other | 3 | 1.0 | 0 | 0.0 |

## S7 Table: Re-interventions

| **Reasons for re-interventions** | **Augmentation** | **No augmentation** |
| --- | --- | --- |
| **Hematoma** | 1 | 3 |
| **Deep wound infection** | 1 | 1 |
| **Lateral blade migration** | 0 | 3 |
| **Excessive screw sliding** | 0 | 2 |
| **Reduction loss** | 0 | 1 |
| **Cutting of screw/blade overall** | 0 | 9 |
| not specified | 0 | 5 |
| Cut out | 0 | 3 |
| Cut through | 0 | 1 |
| **Implant breakage** | 2 | 1 |
|  |  |  |
|  |  |  |
| **Total:** | **4** | **20** |

## Forrest plots

## S1 Fig: Local/device related complications


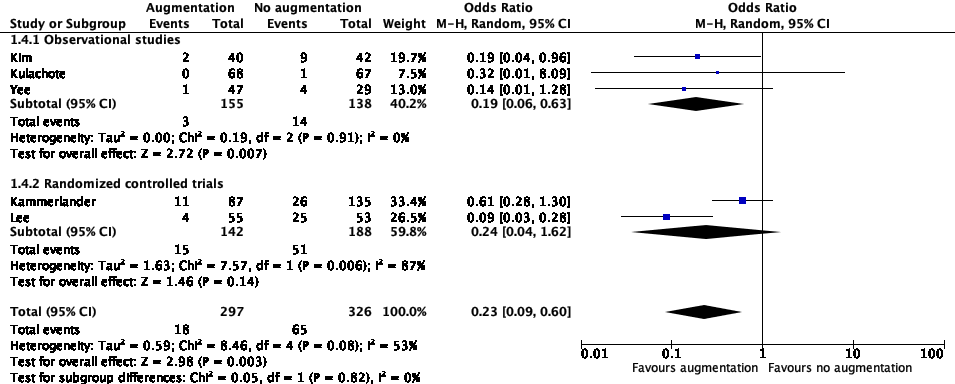


S2 Fig: Systemic complications


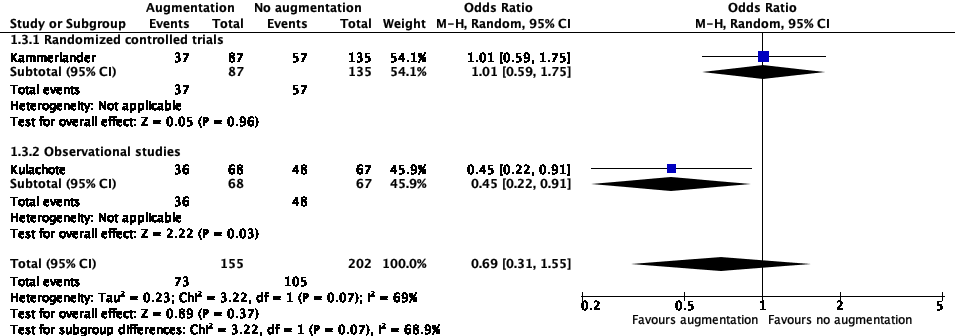


S3 Fig: Thrombembolic events


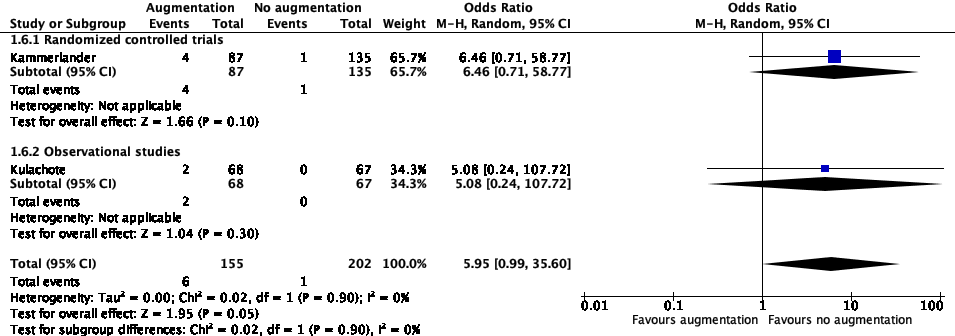


S4 Fig: Mortality


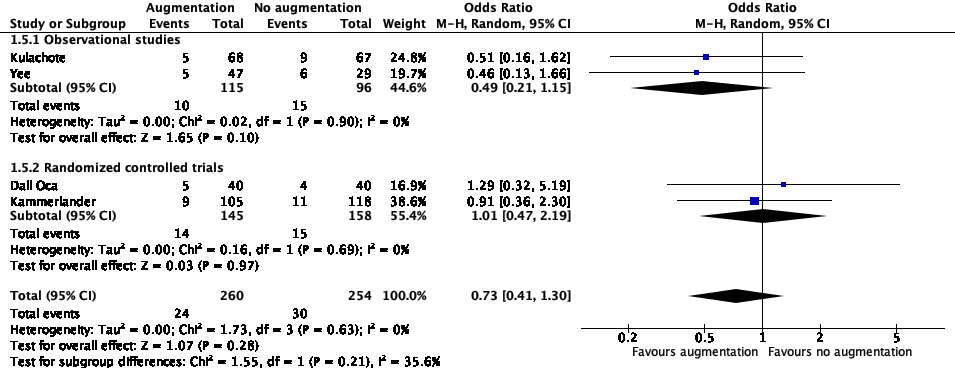


S5 Fig: Duration of hospital stay in days


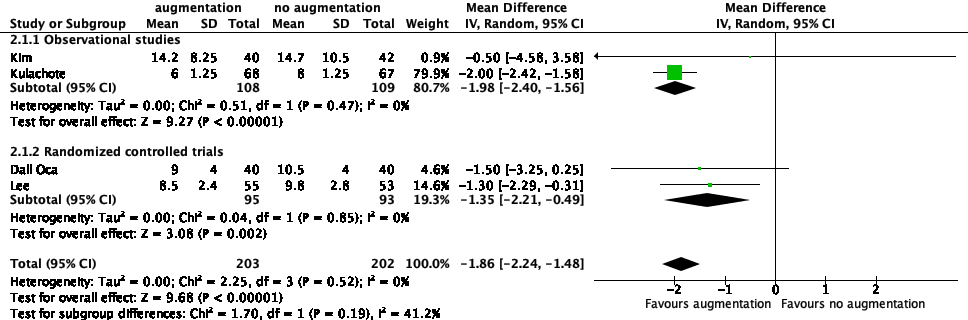


S6 Fig: Operating time in minutes


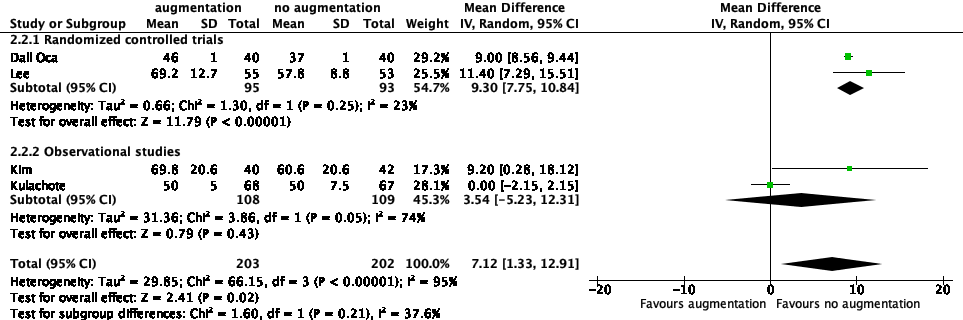


S7 Fig: Sliding of screw/blade


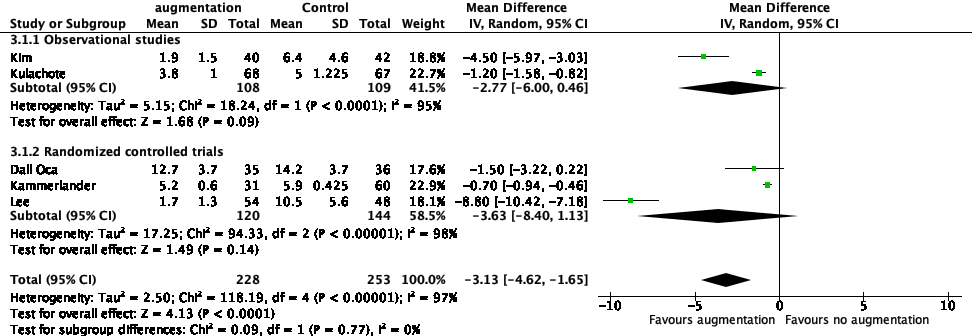


S8 Fig: Varus deviation


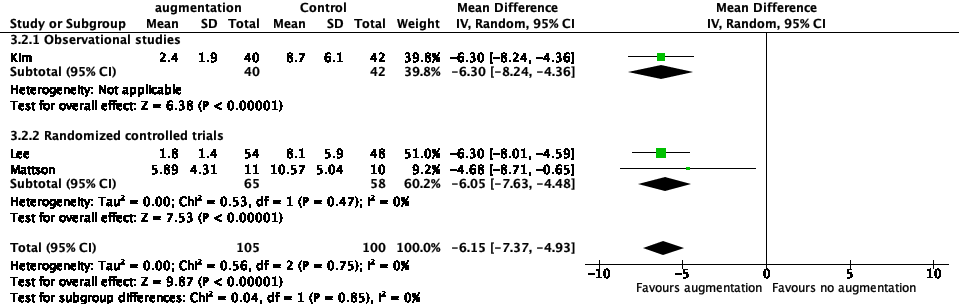


S9 Fig: VAS pain score


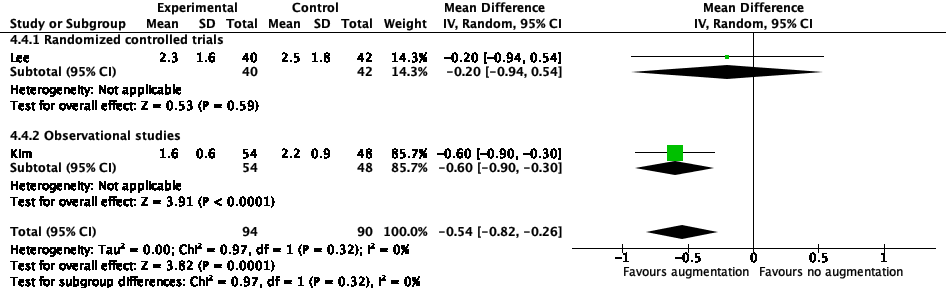


**S10 Fig: Funnel plot overall complications**


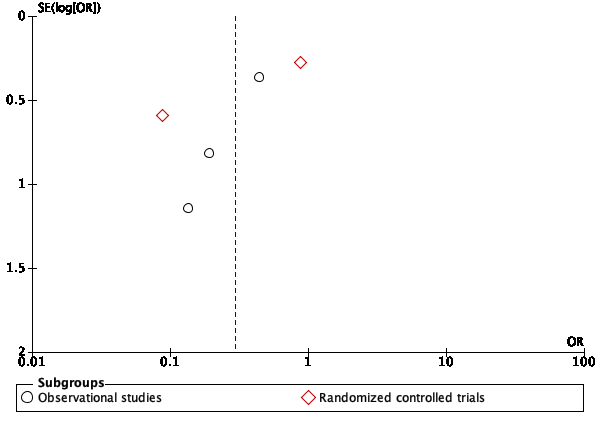


**S11 Fig: Funnel plot local/device related complications**


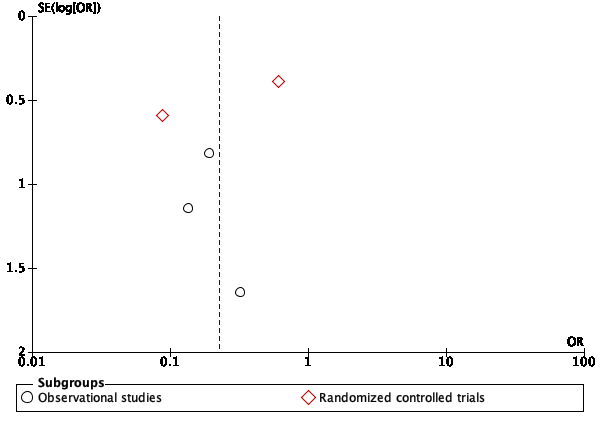


**S12 Fig: Funnel plot mortality**


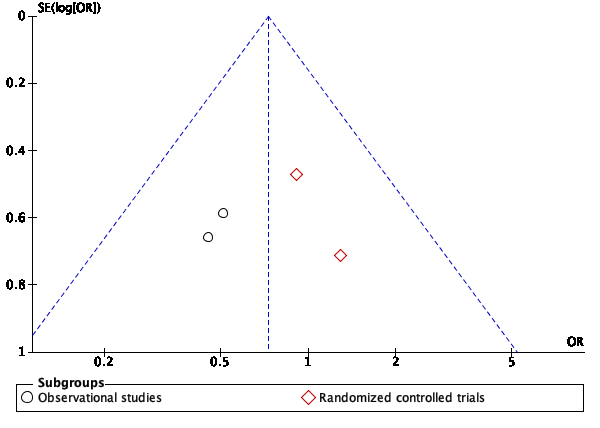


**S13 Fig: Funnel plot duration of hospital stay**


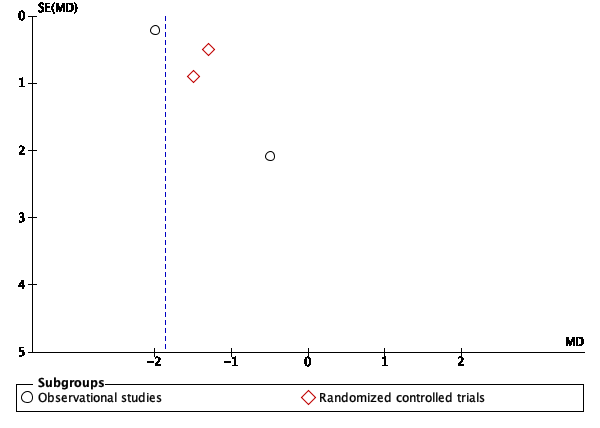


**S14 Fig: Operating time**


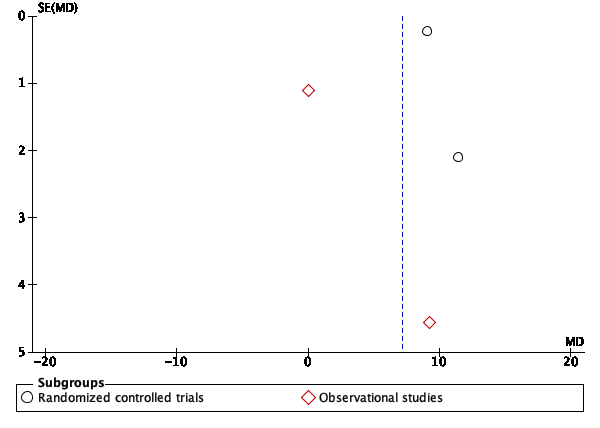


**S15 Fig: Funnel plot Sliding of screw/blade**


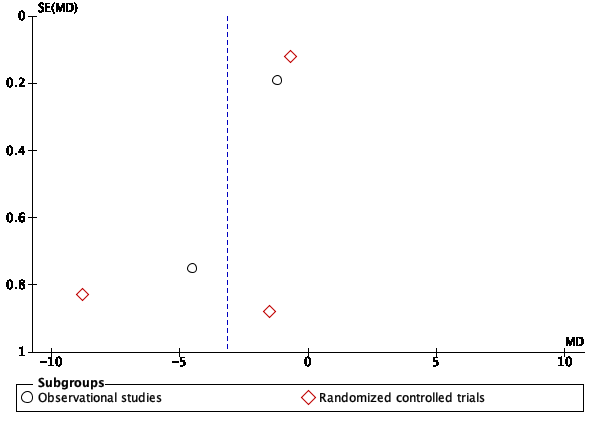


**S16 Fig: Funnel plot varus collapse**


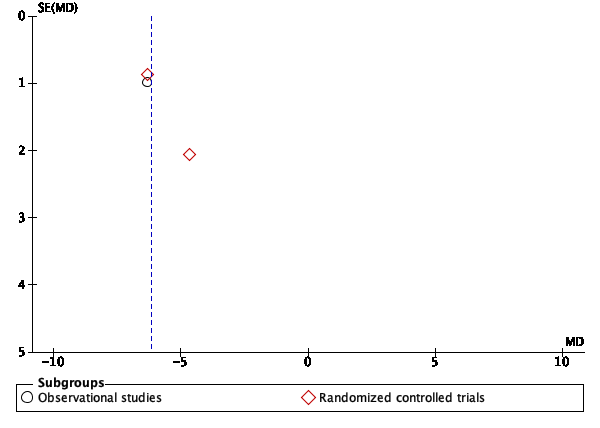


**S17 Fig: Funnel plot functional scores**


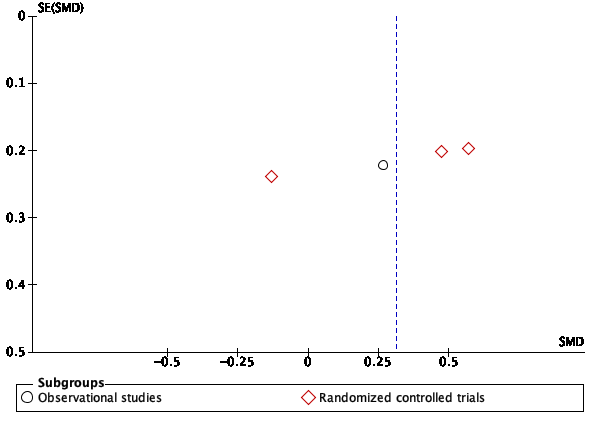

Supplement: S1 File — (DOCX) [file pone.0251894.s002.docx]
